# Efficacy of clofazimine and nitazoxanide combination in treating intestinal cryptosporidiosis and enhancing intestinal cellular regeneration in immunocompromised mice

**DOI:** 10.1016/j.fawpar.2022.e00161

**Published:** 2022-05-04

**Authors:** Marwa Esmat, Amany A. Abdel-Aal, Maisa A. Shalaby, Manal Badawi, Hala Elaskary, Ahmed Badawi Yousif, Mennat-Elrahman A. Fahmy

**Affiliations:** a Department of Medical Parasitology, Faculty of Medicine, Misr University for Science and Technology, 6th October city, Egypt; b Department of Medical Parasitology, Faculty of Medicine, Cairo University, Egypt; c Department of Postgraduate Studies & Scientific Research, Armed Forces College of Medicine (AFCM), Cairo, Egypt; d Medical Parasitology Department, Theodor Bilharz Research Institute (TBRI), Giza, Egypt; e Department of Pathology, National Research center, Giza, Egypt; f Depatment of Medical Parasitology, Faculty of Medicine, Beni-Suef University, Beni Suef, Egypt; g Department of Medical Parasitology, Faculty of Medicine, Fayoum University, Faiyum, Egypt

**Keywords:** *Cryptosporidium*, Clofazimine, Ultrastructure, TEM, Intestinal renewal

## Abstract

*Cryptosporidium* is a widely distributed food and water-borne enteric protozoan that affects a wide range of vertebrates, resulting in life-threatening consequences, particularly in immunocompromised hosts. The lack of effective anti-cryptosporidial drugs may be related to the parasite's unique intestinal location, plus the lack of studies on the process by which the protozoan is able to impair intestinal cellular function. The present work aimed to assess the effect of clofazimine (CFZ), an FDA-approved drug for the treatment of leprosy, as an anti-cryptosporidial drug, using transmission electron microscopy (TEM) and an immunocompromised mouse model. The affected intestinal mucosa with parasitic stages in the infected non-treated group showed signs of severe cellular degeneration, including the loss of tight junctions, deformed and damaged microvilli and irregularly distributed nuclei with a severely vacuolated cytoplasm. Comparatively, nitazoxanide (NTZ) monotherapy showed the lowest efficacy as the drug was associated with the lowest rate of oocyst shedding. In addition, NTZ treatment failed to achieve the return of complete cellular function; abnormalities were evident in the microvilli, cytoplasmic organelles and nuclear features. Clofazimine demonstrated an improvement of the mucosal cellular components, including mitochondria and significantly reduced oocyst shedding. Combined treatment with low-dose CFZ and half-dose NTZ resulted in a significant improvement in the enterocyte cellular structures with an absence of intracellular parasitic stages. These results indicate that CFZ, a safe and readily prescribed drug, effectively reduces cryptosporidiosis when used in combination with only half the dose of NTZ. Used in combination, these drugs were shown to be efficient in regaining intestinal cellular activity following *Cryptosporidium-*induced functional damage in an immunocompromised mouse model.

## 1 Introduction

*Cryptosporidium* is a widely distributed enteric protozoan parasite that infects the gastrointestinal tract of a wide range of vertebrate hosts. In recent years, there has been increasing documentation of its pathogenic role, particularly in pediatric diarrhea (Ashigbie et al., 2021). Infection with *Cryptosporidium* spp. relies primarily on the oral-fecal route of transmission through the consumption of water or food contaminated with infective oocysts (Collinet-Adler and Ward, 2010). Upon ingestion, oocysts excyst and release four sporozoites that can invade the small intestine's enterocytes. In severe cases, the infection may expand beyond the gastrointestinal tract, reaching the respiratory tract as seen in immunocompromised patients (Love et al., 2017).

Currently, 47 species and more than 100 genotypes of the parasite have been reported, with *C. parvum* and *C. hominis* being the most common pathogenic species in humans, responsible for more than 90% of human *Cryptosporidium* infections. (Ahmed and Karanis, 2020; Ježková et al., 2021). The infection with *Cryptosporidium* spp. can cause sickness and severe diarrhea in humans. It can be life-threatening in the very young, the elderly, and immunosuppressed individuals (Chen et al., 2002; Dong et al., 2020). To date, the best therapy for cryptosporidiosis includes fluid and electrolytes replacement, antimotility agents, antiparasitic drugs, nutritional support and reversal of immunosuppression if possible (Checkley et al., 2015; Fahmy et al., 2020; Wang et al., 2020).

Nitazoxanide (NTZ) is the current standard antiparasitic drug used to treat *Cryptosporidium* infection, however, it demonstrates limited and immune-dependent efficacy (Love et al., 2017). The unique cellular location as an intracellular extracytoplasmic protozoan and its capacity for autoinfection can result in the rapid production of numerous oocysts in a relatively short time. Available drugs are not highly effective, and in most cases, will only reduce the duration of shedding. They have little or no effect in treating immunocompromised patients (Thomson et al., 2017; Aboelsoued et al., 2020). Such inadequacy in treatment may also be related to a lack of exploring the mechanisms by which this intestinal protozoan causes impairment of intestinal integrity, especially the role of the epithelium acting as a barrier (Kumar et al., 2018). This level of exploration may require investigation at the cellular ultrastructural level or other more profound forms of studies as opposed to traditionally used histopathological studies.

Recently, a classic drug, clofazimine (Lamprene), has been considered a promising hit compound for cryptosporidiosis (Love et al., 2017; Nachipo et al., 2018; Tam et al., 2020). Clofazimine (CFZ) has been used for more than 50 years to treat leprosy and remains on the World Health Organization (WHO) essential drug list. It is also used as part of a WHO regimen to treat multi-drug resistant *Mycobacterium tuberculosis* with a good safety profile for a condition that requires chronic dosing for a period ranging from 3 to 36 months (Love et al., 2017; Tam et al., 2020). On the other hand, electron microscopy has been used to study parasitic protozoa cytology and micromorphology for at least 40 years and to analyze the host-parasite relationship (Talluri, 1991; de Souza and Attias, 2018). Several studies have reported the ultrastructural features of *Cryptosporidium* spp. stages, the attachment site of the parasite to host cells and their subsequent penetration (Lumb et al., 1988; Talluri, 1991; de Souza and Attias, 2018).

In vivo and in vitro studies showed the development of a dense band and feeder layer after the attachment of sporozoites and merozoites to the host cell, which appears to be unique to the genus *Cryptosporidium* (Current and Reese, 1986; Talluri, 1991; de Souza and Attias, 2018). Another feature of the host-parasite interaction between *Cryptosporidium* and intestinal cells is the development of the parasitophorous vacuole. The parasitophorous vacuole surrounding *Cryptosporidium* is characteristically located at the host's cell surface rather than deep within the cytoplasm (Talluri, 1991; de Souza and Attias, 2018). Further information on cellular ultrastructural changes within the affected epithelium is limited. The present work aimed to assess the effect of CFZ (alone and combined with nitazoxanide) as an anti cryptosporidial chemotherapeutic agent in an immunocompromised mouse model and the use of transmission electron microscopy to study their effect on the host-parasite relationship at the ultrastructural level.

## 2 Materials and methods

### 2.1 Experimental animals and ethical considerations

Female Swiss albino mice of CD1 strain (25–30 g) aged six-eight weeks were purchased from the Animal Center of Theodor Bilharz Research Institute “TBRI”, following the institutional code of ethics for animal research (Approval code: FWA00010609). Mice were maintained in well-ventilated cages with clean wood-chip bedding and provided with ad libitum pelleted food and water at 24 °C under specific pathogen-free conditions.

### 2.2 Induction of immune suppression by dexamethasone

Immune suppression was induced and maintained throughout the experiment by administering dexamethasone (Dexazone, Al Kahira pharmaceutical, and chemical industries company) orally by using oral-gastric gavage at a dose of 0.25 mg/g/day for 14 successive days prior to inoculation with *Cryptosporidium* oocysts (Rehg et al., 1988).

### 2.3 Preparation and inoculation of infection

*Cryptosporidium parvum* oocysts were isolated from the feces of a naturally infected diarrheic calf at the veterinary clinic, Faculty of Veterinary Medicine, Cairo University. A sample was stained with Kinyoun's Acid Fast stain to identify *Cryptosporidium* (Operario et al., 2015). The discontinuous sucrose density gradient flotation technique was used (Arrowood and Sterling, 1989) to concentrate and purify the oocysts. The oocysts were then suspended in PBS and stored in two volumes; one at −20 °C for molecular study, and the other kept at 4 °C for infecting mice contained 0.01% Tween-20, 200 IU/ mL penicillin, 0.2 mg/mL streptomycin, and 2.5 μg/mL Amphotericin B to inhibit bacterial or fungal growth.

Identification of the parasite using the PCR-Restriction Fragment Length Polymorphism (RFLP) of the small subunit (SSU) rRNA gene method of Xiao et al., 2001 was performed by Dr. Mohamed Sharaf Badr, Department of Molecular Biology, Medical Research Center, Faculty of Medicine, Ain Shams University. Mice in infected groups were inoculated by oral-gastric gavage with 1 × 10^4^ *C. parvum* oocysts (Love et al., 2017).

### 2.4 Study groups

Mice were classified into four groups of immunosuppressed (6 mice/group) (Huang and Yang, 2002; Fahmy et al., 2021) Group A: infected only; Group B: infected and treated with NTZ; Group C: infected and treated with CFZ; Group D: infected and treated with half-dose nitazoxanide + half-dose clofazimine.

### 2.5 Drugs

NTZ was supplied as suspension 100 mg/ 5 mL (Nanazoxid; from Medizen Pharmaceutical Industries for Utopia Pharmaceuticals). Each mouse in group B, 250 mg/kg/day was administered orally once daily from the fourth-day post-inoculation for ten successive days while a dose of 125 mg/kg/day was used for group D (Theodos et al., 1998; Fahmy et al., 2020).

CFZ was supplied as soft gelatin capsules 100 mg (lamprene; from Novartis pharmaceuticals Corporation, New Jersey). Each mouse in group C, 10 mg/kg/day was administered orally once daily from the fourth-day post-inoculation for three successive days while a dose of 5 mg/kg/day was used for group D (Love et al., 2017).

### 2.6 Parasitological analysis

Fresh fecal pellets from the infected mice were collected from the 3rd day post-inoculation (pi) and every three days until the end of the experiment (30 days) (Cui et al., 2018), tested for oocysts using the formol/ether centrifugal sedimentation method. Kinyoun's Acid-Fast stain (cold method) was used to detect and estimate the number of oocysts per gram of feces in 50 μL (Smith, 2008; Benamrouz et al., 2012).

### 2.7 Histopathological and ultrastructural examination

At the end of the experiment, the terminal ileum of each mouse was removed, processed by standard methods and stained for histopathology using hematoxylin and eosin. At necropsy, 1mm^3^ pieces of the small intestine of each mouse were removed and fixed in 2% glutaraldehyde in 0.1 phosphate buffer, postfixed in 1% osmium tetraoxide for 2 h at 4 °C, dehydrated, and embedded in epon. Semi-thin and ultrathin sections from 2 samples/each mouse were prepared using Ultramicrotome (Leica Ultracut R). Ultrathin sections were stained with uranyl acetate and lead citrate. The sections were analyzed by transmission electron microscope (JEOL-Ex 1010 transmission electron microscope at Al-Azhar University).

### 2.8 Statistical analysis

Results were presented as the mean and standard deviation (SD). Data were analyzed by STATA/IC software version 16.1 (Stata Corp., Lakeway, TX, USA). One-way analysis of variance (ANOVA) and post hoc tests were done for multiple comparative analyses between the study groups. *P*-values <0.05 were considered statistically significant.

## 3 Results

### 3.1 Effect of clofazimine on oocyst shedding

**Fig. 1:**
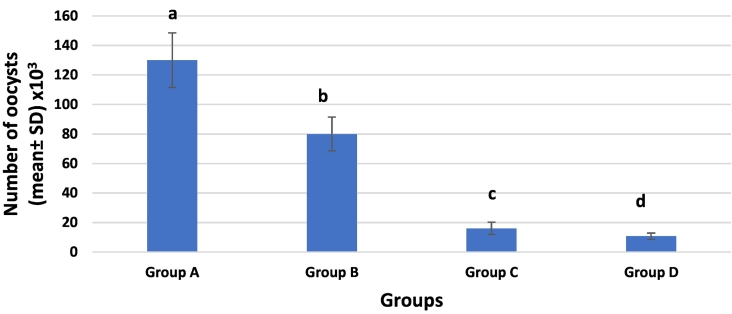
Bar chart shows *Cryptosporidium* oocysts shedding/g feces in all groups (A: infected only; B: infected and treated with NTZ; C: infected and treated with CFZ; D: infected and treated with half-dose nitazoxanide + half-dose clofazimine).

There was a significant difference (*p* < 0.05) between all groups when comparing the number of oocysts shed per gram of feces in treated groups with the positive control group at the end of the experiment (Fig. 1). Oocyst shedding was highest in the group treated with NTZ only (80,000 oocysts/g) with a reduction rate of only (38%) when compared with the low shedding rate in the group treated with CFZ alone (16,000 oocysts/g) with a reduction rate of (87.7%). While the group treated with NTZ & CFZ combination showed the lowest shedding rate (10,700 oocysts/g) and the highest percentage of reduction (91.8%).

### 3.2 Ultrastructural characteristics of host-parasite interactions after clofazimine treatment

**Fig. 2:**
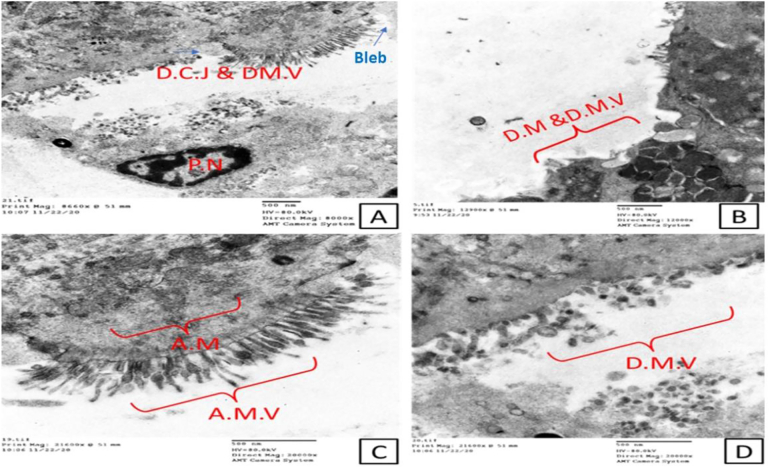
Electron micrograph shows changes in the infected non-treated mucosal lining A; Completely damaged epithelium lining, including cellular junction which shows discontinuity and damage of microvilli (D.C.J & D.M.V). Also, notice the enterocyte apical blebbing (blue arrows). The pyknotic nucleus appears within the section. B; severely damaged fragmented mitochondria (D.M) with many lysosomes with damaged atrophied villi. C; Abnormal microvilli with swollen end and abnormal mitochondria within the cytoplasm with ballooning to some of them and complete damage to others. Abbreviations: D.C.J; Discontionus Cellular Junction, D.M.V; damaged microvilli, DM for Damaged Moitochondria, AMV; Abnormal Microvilli and AM for Abnormal Mitochondria (Scale bar; 500 nm). (For interpretation of the references to colour in this figure legend, the reader is referred to the web version of this article.)

**Fig. 3:**
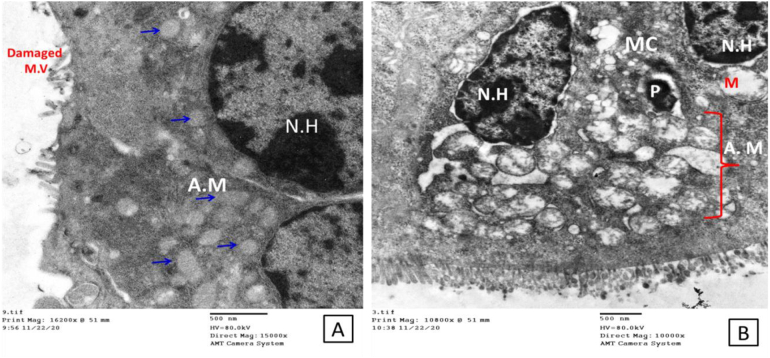
Electron micrograph shows changes in the infected non-treated mucosal lining. A; nuclei with a lot of heterochromatin (N.H), damaged microvilli (M.V), and a lot of abnormal mitochondria (A.M; blue arrows). B; Microfold cells (M.C) containing internalized parasite (P) notice the untightly arranged microvilli (Scale bar; 500 nm). (For interpretation of the references to colour in this figure legend, the reader is referred to the web version of this article.)

**Fig. 4:**
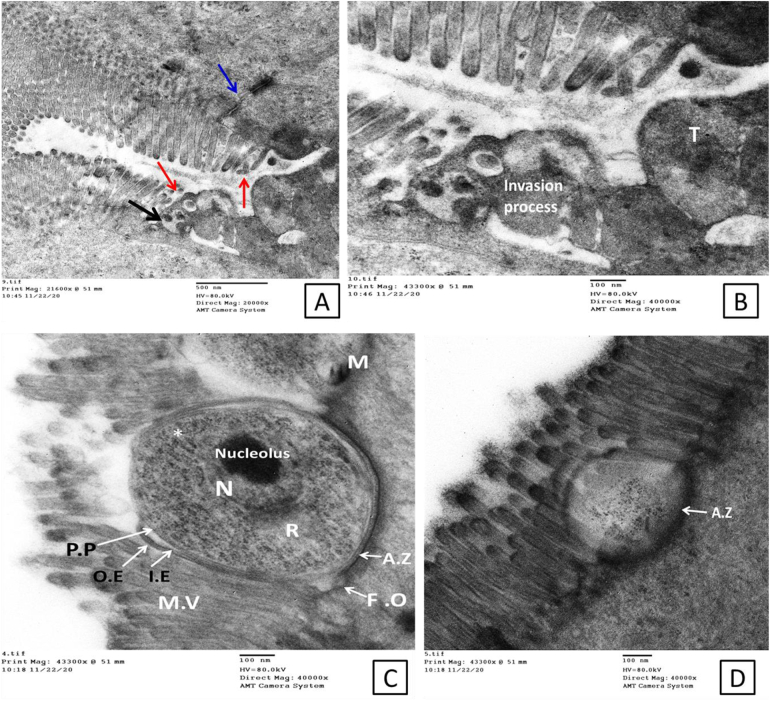
Electron micrographs show some of the parasitic stages within the brush borders of the affected intestinal mucosa. A; thin-walled sporulated oocysts (black arrow), about to release their contents to invade another cell. Notice the affected microvilli within the affected area (red arrows) and the interrupted tight junction (blue arrow). B; larger view shows the invasion process with a nearby developed trophozoite with an evident nucleolus. C; another parasitic stage with apparent internal and external features shows attachment zone (A.Z), a dense band subjacent to the parasite-absorptive cell interface (AC), starting feeder organelles (F.O), pellicle of the parasite (P.P), outer and inner envelops (O.E & I.E), forming endoplasmic reticulum (*) plus Electron-lucent appearance of the rhoptries (R) (Scale bar; 500 nm in A and 100 nm in B, C &D). (For interpretation of the references to colour in this figure legend, the reader is referred to the web version of this article.)

Transmission electron microscopy of the intestinal epithelium of the infected non-treated control group showed morphological abnormalities in the form of severe disarrangement, distortion and blunting of the microvilli that form the brush border (Fig. 2). Enterocytes showed destructive changes such as abnormal nuclei and numerous swollen mitochondria (Fig. 3). The ileal epithelial cells of immunocompromised infected non-treated mice displayed a significantly damaged epithelial lining with abnormally disfigured (swollen ends) and/or damaged atrophied microvilli. Cellular junctions showed discontinuity with some pyknotic nuclei (Fig. 2A&C). Mitochondria displayed many abnormalities; swollen, severely damaged, or fragmented with many lysosomes (Fig. 2B&C and Fig. 3A). Nuclei with a large amount of heterochromatin were observed (Fig. 3A). Microfold (M) cells containing internalized parasites are shown in Fig. 3B. *Cryptosporidia* stages were seen in the affected areas. Enlargement of the nucleus and nucleoli and the development of a zone with interdigitated membranous folds at the host-parasite interface were observed (Fig. 4).

**Fig. 5:**
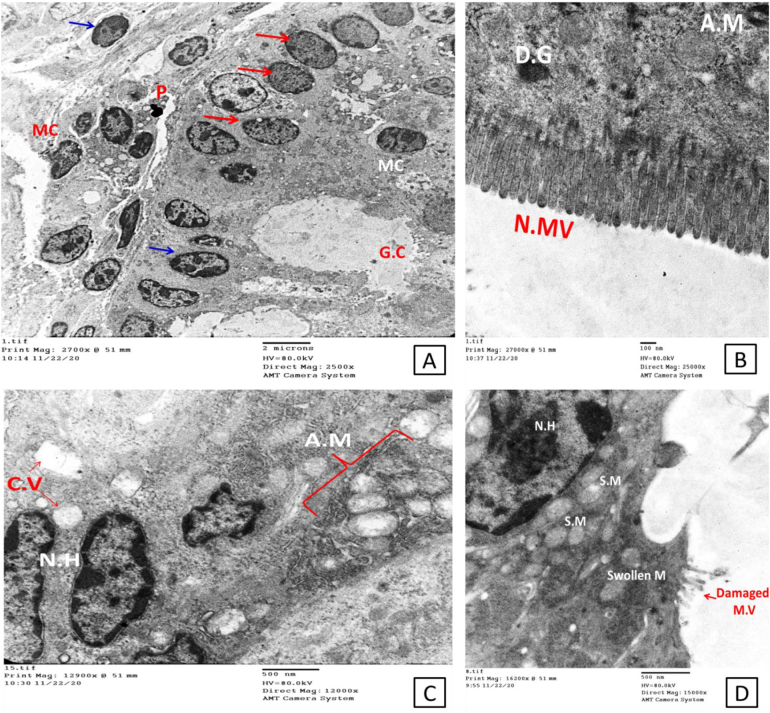
Electron micrograph shows the status of the mucosal lining with signs of incomplete cellular recovery with signs of non-efficacious renewal in the group treated with NTZ. Some nuclei with euchromatin are seen in A (red arrows), still with an irregular basal distribution. Other nuclei, rich in heterochromatin (N.H), are still seen in A (blue arrows), C & D, as well as abnormal swollen mitochondria with areas of degenerated damaged microvilli in B. Cytoplasmic vacuolation is seen in C. C; Other areas with apparently healthy microvilli, yet with few abnormal cytoplasmic mitochondria and dense granules (Scale bar; 2 μm in A, 500 nm in C&D and 100 nm in B and). (For interpretation of the references to colour in this figure legend, the reader is referred to the web version of this article.)

**Fig. 6:**
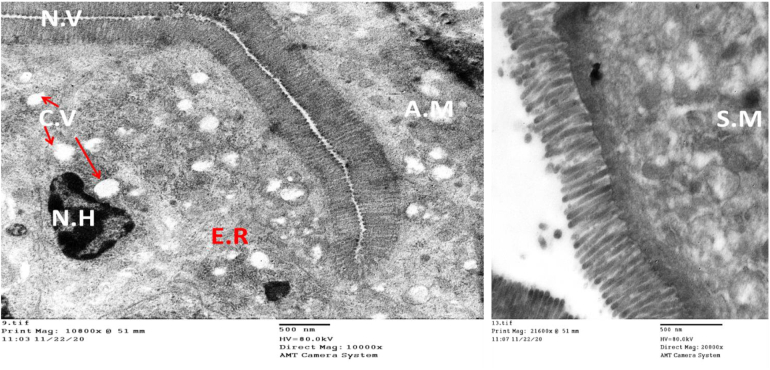
Electron micrograph of the group treated with CFZ shows some improvement concerning cellular renewal with normal healthy microvilli and regain of some cellular function as rough and smooth endoplasmic reticulum appeared, yet with few areas of cytoplasmic vacuolation (CV) and abnormal or swollen mitochondria (AM & SM), possibly related to mitophagy (Scale bar; 500 nm).

The epithelial lining showed improvement in the group treated with nitazoxanide compared to the control infected non-treated group. However, the mucosal lining showed signs of incomplete cellular recovery and non-efficacious cellular renewal. Some nuclei with euchromatin were seen with an irregular basal distribution. In addition, nuclei rich in heterochromatin were identified (Fig. 5A). Furthermore, abnormal enlarged mitochondria with areas of degeneration were observed (Fig. 5C&D). Cytoplasmic vacuolation was also observed with apparently healthy microvilli, yet with abnormally cytoplasmic mitochondria and dense granules (Fig. 5C), damaged microvilli were also observed not sure if this makes sense as you just said they were apparently healthy? (Fig. 5B). Ultrastructural examination of the ileal regions of mice treated with CFZ revealed a significant amount of normal ultrastructure with very few areas of cytoplasmic vacuolation and abnormal mitochondria (Fig. 6).

**Fig. 7:**
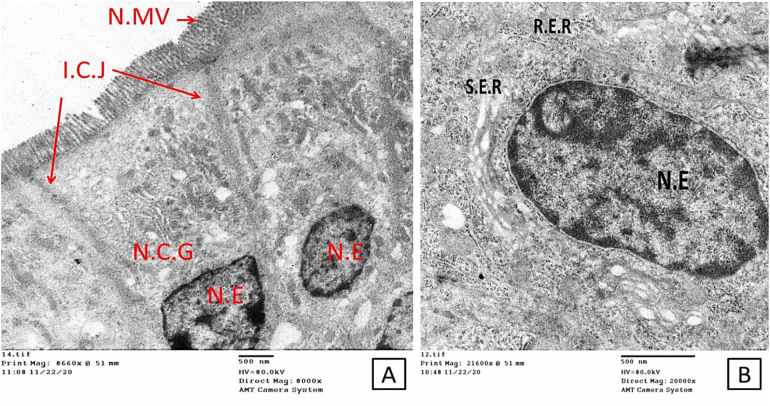
Electron micrograph shows the dramatic improvement in the mucosal lining with signs of successful cellular regeneration and renewal. A; nuclei with a lot of Euochromatin (N.E), healthy normal microvilli (N.MV) and intact cellular junction (I.C.J), and normal cytoplasmic organelles (N.C.G). B; larger view to clarifying recovery on cellular and organellar levels; nucleus with Euchromatin, surrounded by rough and smooth endoplasmic reticulum (R.E.R & S.E.R), indicating cellular functional recovery (Scale bar; 500 nm).

Ultrastructural examination of the ileal regions of mice from the group treated with combined therapy revealed the best results; the mucosal lining showed signs of successful cellular regeneration and renewal and was confirmed by the absence of *Cryptosporidium* stages compared to the infected non-treated group. Complete repair of the brush border with parallel tightly adherent microvilli was evident. A small number of tips of the microvilli were covered with glycocalyx, the enterocytes were typically ovoid, with basally located nuclei containing prominent nucleoli and normal chromatin. The cytoplasm of the absorptive cells contained typical mitochondria with well-developed cristae. Rough and smooth endoplasmic reticulum (ER) were seen in the cytoplasm (Fig. 7).

## 4 Discussion

In the present study, the efficacy of the antileprotic FDA-approved drug, CFZ was assessed as an anti-cryptosporidial agent to treat cryptosporidiosis in experimentally infected mice. The drug alone reduced the infection significantly by 87.7% and improved the pathological ultrastructural cellular changes caused by cryptosporidiosis in the ileal regions of infected immunosuppressed mice. When used in combination with a half dose of NTZ, CFZ showed a significant reduction rate of 91,8% and efficient improvement of the ultrastructural abnormalities, significantly more than the reference drug; NTZ alone or CFZ alone. In vivo reduction of oocyst shedding <1% of the control group within 24 h of the end of CFZ treatment has been previously reported by Love et al. (2017). However, Tam et al. (2020) stated that CFZ couldn't clear *Cryptosporidium* parasites or improve clinical symptoms in human immunodeficiency virus (HIV) patients with cryptosporidiosis. Moreover, it may have worsened the diarrhea episodes. This could be due to improper adjustment of the dose and lack of effect on other pathogens (Nachipo et al., 2018; Zhang et al., 2021).

Our study identifies the ultrastructural alterations of the intestinal epithelium, particularly enterocyte cells and their constituents, such as mitochondria, lysosomes, endoplasmic reticulum, tight junctions and microvilli in the infected non-treated group. The microvilli of the immunosuppressed infected non-treated group showed severe disarrangement, distortion, and blunting. Disruption of the epithelium and disintegration of villi following enteric infections were reported (Scott et al., 2002; Cooper et al., 2013; Shini et al., 2021). Disruption of the tight junctions of the epithelium and cytoplasmic vacuolation were also observed in the intestinal mucosa of the infected mice. Previous studies reported changes in the morphology and functionality of tight junctions with the fragmentation of intercellular junctional complexes and increased cytoplasmic vacuolization accompanying enteric pathogens and described them as features of ongoing necrosis (Olkowski et al., 2008; Guttman and Finlay, 2009; Leite et al., 2013; Shubin et al., 2016). We identified ‘bleb formation’ on the enterocyte apical membrane in the infected non-treated group, this is in agreement with the study conducted by Chen et al. (1997) who reported that rabbit renal proximal epithelial cells formed extensive blebs after hypoxic injury. Blebbing was initially assumed to be related solely to pathological conditions in response to nonspecific cellular insults. However, it is now considered an essential physiological process that occurs during cell blastulation, viral infection, protective mechanisms against injury (either physical or chemical stress) and as a hallmark of the execution stage of apoptosis (Khajah and Luqmani, 2016). The most striking finding in our results within the control infected non-treated group was the significant abnormality in the shape and disruption of the mitochondria. Mitochondria play an essential role in cellular homeostasis and innate immunity by their existing regeneration balanced nature between fission and fusion in response to intracellular or extracellular incentives (Ma et al., 2006; Han et al., 2018). Microbial infections, including *Cryptosporidium*, negatively affect this mitochondrial balance, decreasing the capacity of these vital organelles to generate the energy needed for healthy cellular survival. Therefore, normal mitochondria are required for normal cellular regeneration, any abnormal features that affect the known mitochondrial dynamics are considered signs of cellular toxicity and subsequent cellular death (McKay et al., 2020). Drugs that can stabilize mitochondrial dynamics and counteract the effect of the microbial agent on such vital organelles may be considered efficient candidate drugs for treating infectious diseases affecting the mucosal surfaces.

Concerning M cells containing internalized parasites detected in the infected non-treated group, the gastrointestinal tract's epithelial lining contains specialized cells that are believed to act as specific antigen transport cells within a process called “transcytosis”. This specific transcellular activity facilitates exposing the microbial immunogen to the local lymphoid follicles thereby stimulating the immune response. *Cryptosporidium* parasitic stages are among the first parasitic models identified within the M cells. This way of entry likely allows antigenic recognition by the intestinal immune system and hence represents a vital element of mucosal immunity that helps in eradicating infection even in immunologically altered hosts. This may explain why *Cryptosporidium* infection is self-limiting in immunocompetent cases via such unique M cells (Marcial and Madara, 1986; Kucharzik et al., 2000). Another observation was nuclear pyknosis, also known as karyopyknosis, which is a sign of necrosis. The chromatin became irreversibly condensed and was followed by fragmentation or what is called karyorrhexis (Kroemer et al., 2009). All these cellular ultrastructural changes verified the powerful negative effect of the infection on intestinal cellular function, regardless of the dexamethasone administration as Fujino et al. (1996) reported that there were no marked ultrastructural differences between the intestinal epithelial cells from the untreated control and dexamethasone-treated mice.

Combination therapy in this study generated the best results regarding the complete recovery of enterocytes with typical features. NTZ monotherapy failed to positively impact the intestinal cellular function, this may explain its repeated documented failure in treating such protozoal infections in immunocompromised cases. In our work, we used NTZ as the positive control drug. Nitazoxanide is currently the only drug approved by the FDA to treat cryptosporidiosis in humans and the most frequently studied drug with subsequent effectiveness against *C. parvum* in suckling mice, nude mice, gerbils, rats and piglets (Blagburn et al., 1998; Theodos et al., 1998; Baishanbo et al., 2005; Gargala, 2008; Checkley et al., 2015; Diptyanusa and Sari, 2021). Paromomycin was also used to treat mice infected with cryptosporidiosis and significantly lowered the oocyst shedding. However, it had little efficacy against infections in inaccessible sites such as the pits of the pyloric region of the stomach and the cecum where most of the infected crypts were distorted. The guts of infected mice treated with paromomycin remained heavily infected (Tzipori et al., 1994; Theodos et al., 1998). In this study, we used a combination of half the dose of CFZ with half the dose of NTZ. This was based on observations from previous studies of Tuvshintulga et al. (2021) who reported excellent efficacy and complete clearance of *Babesia microtti* infection after 44-days of treatment with a combination of half dose of CFZ and atovaquone in immunocompromised mice. Furthermore, Ammerman et al. (2018) used a half dose of CFZ to add potent bactericidal and treatment-shortening activity to the first-line regimen in *M. tuberculosis*-infected BALB/c mice. In addition, studies by Ruth et al. (2019) reported a synergistic interaction between bedaquiline and CFZ combination therapy against *Mycobacterium abscessus.* We used an immunocompromised mouse model in our study. They are considered the gold standard for in vivo *Cryptosporidium* infection studies as they provide the unique dynamic environment of the host intestine with its complex tissue architecture, diversity of cell types and host immune responses. However, they have limitations due to the inability to adequately manipulate these host organisms' genetics, the high costs associated with their upkeep and the absence of diarrhea (Theodos et al., 1998; Marzook and Sateriale, 2020).

In conclusion, our findings provide new insights into the pathogenesis of cryptosporidiosis and the effects of single and combined treatments. NTZ alone showed the lowest efficacy in treating the infection, as the drug had the lowest oocyst reduction rate. In addition, the drug seemed unable to regain complete cellular function as was evidenced by the abnormal features of the microvilli, cytoplasm, nuclei and other cellular organelles. CFZ treatment resulted in more significant improvements in the mucosal cellular components and a higher oocyst reduction rate. Interestingly, combined treatment with CFZ and half dose of NTZ resulted in the highest reduction rate of shed oocysts, hence, the observed improvement in the enterocyte structure. Future studies should be conducted to determine the synergistic effect of both drugs.

## Declaration of Competing Interest

The authors whose names are listed immediately below certify that they have NO affiliations with or involvement in any organization or entity with any financial interest (such as honoraria; educational grants; participation in speakers' bureaus; membership, employment, consultancies, stock ownership, or other equity interest; and expert testimony or patent-licensing arrangements), or non-financial interest (such as personal or professional relationships, affiliations, knowledge or beliefs) in the subject matter or materials discussed in this manuscript.
